# Identification and characterization of a plastidial ω-3 fatty acid desaturase *EgFAD8* from oil palm (*Elaeis guineensis* Jacq.) and its promoter response to light and low temperature

**DOI:** 10.1371/journal.pone.0196693

**Published:** 2018-04-26

**Authors:** Lizhi Chen, Lei Wang, Herong Wang, Ruhao Sun, Lili You, Yusheng Zheng, Yijun Yuan, Dongdong Li

**Affiliations:** 1 Key Laboratory of Advanced Materials of Tropical Island Resources, Ministry of Education; Department of Bioengineering, College of Material and Chemical Engineering, Hainan University, Haikou, Hainan, China; 2 Institute of Tropical Agriculture and Forestry, Hainan University, Haikou, Hainan, China; Huazhong University of Science and Technology, CHINA

## Abstract

In higher plants, ω-3 fatty acid desaturases are the key enzymes in the biosynthesis of alpha-linolenic acid (18:3), which plays key roles in plant metabolism as a structural component of both storage and membrane lipids. Here, the first ω-3 fatty acid desaturase gene was identified and characterized from oil palm. The bioinformatic analysis indicated it encodes a temperature-sensitive chloroplast ω-3 fatty acid desaturase, designated as *EgFAD8*. The expression analysis revealed that *EgFAD8* is highly expressed in the oil palm leaves, when compared with the expression in the mesocarp. The heterologous expression of *EgFAD8* in yeast resulted in the production of a novel fatty acid 18:3 (about 0.27%), when fed with 18:2 in the induction culture. Furthermore, to detect whether *EgFAD8* could be induced by the environment stress, we detected the expression efficiency of the *EgFAD8* promoter in transgenic Arabidopsis treated with low temperature and darkness, respectively. The results indicated that the promoter of *EgFAD8* gene could be significantly induced by low temperature and slightly induced by darkness. These results reveal the function of EgFAD8 and the feature of its promoter from oil palm fruits, which will be useful for understanding the fuction and regulation of plastidial ω-3 fatty acid desaturases in higher plants.

## Introduction

Linolenic acid (18:3) plays important roles in plant metabolism as a structural component of storage and membrane lipids, and as a precursor of signaling molecules involved in plant development and stress response [1,2]. For human, alpha-linoenic acid (18:3 ω-3) is one of essential fatty acids, which are necessary for health and must be acquired from diet [3]. Moreover, 18:3 is a metabolic precursor for long-chain polyunsaturated fatty acids (LC-PUFAs) synthesis [4], such as eicosapentaenoic acid (EPA; 20:5Δ^5,8,11,14,17^, ω-3) and docosahexaenoic acid (DHA; 22:6Δ^4,7,10,13,16,19^, ω-3). They are essential constituents of human nutrition, and play key roles in brian and cardiovascular health [5–8]. However, the primary source of EPA and DHA is the marine product, especially fish oil [9].

In higher plants, fatty acids are synthesized *de novo* in the stroma of plastids through a complex series of condensation reactions to produce either C16 or C18 fatty acids. These fatty acids are then incorporated into the two glycerolipid synthetic pathways: the prokaryotic pathway and eukaryotic pathway [10,11]. Chloroplast galactolipids contain substantial amounts of trienoic fatty acid, either the hexatrienoic acid (16:3) or 18:3, which may vary in different plant species [12]. In both glycerol pathways, desaturation of fatty acids is performed by a series of membrane-bound desaturases of the chloroplast and the endoplasmic reticulum (ER) [2,11].

Fatty acid desaturases are encoded by nuclear genes and differ in the substrate specificity and subcellular localization, and play critical roles in maintaining proper structure and cellular function of biological membranes [2,13,14]. Stearoyl ACP desaturase (SAD) is the only soluble desaturase, which convertes stearoyl-ACP (18:0-ACP) to oleic acid (18:1-ACP) by introducing the first double bond at the 9^th^ position from the carboxylic end of the fatty acyl chain in the process of fatty acid synthesis [14,15]. Furhter desaturations are carried out by membrane-bound desaturases in the ER (FAD2 and FAD3) and the chloroplast (FAD4, FAD5, FAD6, FAD7, and FAD8) as we mentioned before. FAD2 and FAD6 are ω-6 desaturases that synthesize linolenic acid (18:2) from oleic acid (18:1) by introduction of a double bond at the 12^th^ position from carboxylic end or 6^th^ position from the ω end in the ER and plastid, respectively. While, FAD3, FAD7, and FAD8 are ω-3 desaturases that desaturase 18:2 to 18:3 by inserting a double bond at the 15^th^ position from the carboxylic end or 3^rd^ position from the ω end. Moreover, the *FAD8* gene encodes a plastidial ω-3 desaturase that is cold-inducible, which can compensate the decreased expression of *FAD7* under the low temperature [16,17]. FAD4 and FAD5 synthesize 16:1 from 16:0 specifically for the main component of chloroplast membrane phosphatidylglycerol and monogalactosyldiacylglycerol, respectively [15].

The genes encoding ω-3 fatty acid desaturases have been identified and studied from several plant species, such as Arabidopsis [16,18,19], soybean [11,13,20], *Zea mays* [21], olive [1,22], safflower (*Carthamus tinctorius* L.) [23], *Jatropha curcas* [24], and flax [25]. Microsomal FAD3 enzymes have been shown to be the major contributors to seed 18:3 content in Arabidopsis, soybean, and flax [14,18,20,25]. On the contrary, the overexpression of a chloroplast ω-3 fatty acid desaturase was demonstrated to enhance the tolerance to low temperatures [26,27]. Furthermore, overexpression of ω-3 fatty acid desaturases *BnFAD3* from *Brassica napus* and *StFAD7* from *Solanum tuberosum* in tomato enhanced resistance to cold stress, and altered fatty acid composition with an increase in the 18:3/18:2 ratio in leaves and fruits [28]. Increased desaturation of glycerolipids severs as compensation to cold-caused decrease in membrane fluidity [29,30]. Thus, plant desaturases are very sensitive to several environmental cues such as temperature, light, or other factors [11,31].

Oil palm (*Elaeis guineensis* Jacq.) is the most productive oil-bearing crop in the world [32]. It is native to West Africa, but is now grown thuoughout the humid tropical lowlands [33,34]. There are two major economic productions from oil palm fruits: palm oil extracted from the mesocarp and palm kernel oil from the endosperm [35,36]. Palm oil contains oleate (18:1; 45%), linoleate (18:2; 8%), and 47% saturated fatty acid (43% of 16:0 and 4% of 18:0). Therefore, much effort has been made to understand the oil synthesis in oil palm fruits and improve the fatty acid composition of palm oil since the high content of saturated fatty acid has been thought to be unhealthy to human [37]. The activity of a Δ12 fatty acid desaturase from oil palm has been tested in yeast, and it produced about 12% of linoleic acid in yeast, which barely exist in wild-type yeast cells [38]. Recently, the *in vivo* function of a *DGAT2* gene from oil palm mesocarp has been verified in yeast and transgenic Arabidopsis, exhibiting a substrate preference towards unsaturated fatty acids [39].

In the present work, a chloroplast ω-3 fatty acid desaturase (*EgFAD8*) and its promoter from oil palm has been isolated and characterized. The expression pattern of *EgFAD8* in leaves and mesocarp at five developmental stages has been detected by Real-time quantitative PCR. The *in vivo* function of EgFAD8 has been verified by overexpression in *Saccharomyces cerevisiae* (INVSc1) with exogenous linoleic acid (18:2). To detect whether it could be induced by low temperature or the change of environment, we detected the efficiency of the *EgFAD8* promoter in transgenic Arabidopsis treated with low temperature and darkness, respectively. These results revealed the function of *EgFAD8* and the feature of its promoter from high plants, which will be useful for understanding the role of ω-3 fatty acid desaturases from fruits grown in the tropical areas.

## Materials and methods

### Materials

#### Ethics statement

No specific permissions were required for these locations/activities. We confirmed that the field studies did not involve endangered or protected species.

Oil palm fruits were collected from the Coconut Research Institute, Chinese Agricultural Academy of Tropical Crops, Wenchang, Hainan, China. Fruits at five developmental stages (30–60 days after pollination (DAP), 60–100 DAP, 100-12- DAP, 120–140 DAP, and 140–160 DAP) were immediately frozen in liquid nitrogen and stored at -80°C until use. Wild-type *Arabidopsis thaliana* ecotype Columbia was used in this study. Arabidopsis plants were cultivated in a growth chamber at 23°C with a 16-h photoperiod (16 h of 150 μE m^-2^ sec^-1^ light and 8 h of darkness). Yeast (*Saccharomyces cerevisiae*) strain INVSc1 (MAT**a** *his3*Δ*1 leu2 trp1-289 ura3-52*/MATα *his3*Δ*1 leu2 trp1-289 ura3-52*) and the high copy number shuttle vector pYES2 (Invitrogen, USA) were used for gene function analysis.

### Bioinformatic analysis

Nucleotide and amino acid sequence analyses used the BLAST program and open reading frame (ORF) finder from NCBI website (https://www.ncbi.nlm.nih.gov/). Amino acid alignment of fatty acid desaturases was performed by ClustalX 2.1 program with default setting [40]. A phylogenetic tree was constructed using the neighbor-joining method in MEGA5 [41]. Promoter prediction and *cis*-acting regulatory element analysis were carried out on the PlantCARE (http://bioinformatics.psb.ugent.be/webtools/plantcare/html/) [42].

### Cloning of *EgFAD8* gene

Total RNA from mesocarp was extracted by cetyltrimethylammonium bromide (CTAB) based method as described previously [43]. First-strand cDNA was synthesized from 1 μg of total RNA using FastQuant RT Kit (Tiangen, Beijing, China) according to manufacturer’s instructions, and was used for gene cloning and expression analysis. The coding sequence of EgFAD8 was amplified by Phusion High-Fidelity DNA Polymerase (Thermo Fisher Scientific, USA) using primers EgFAD8-pYES2 F and R with restriction site *Kpn*I and *Xba*I, respectively (Table 1). PCR was performed with an initial denaturation at 98 °C for 30s, 30 cycles at of 98 °C for 10 s, 56 °C for 30s, 72 °C for 60 s and a final extension step of 72 °C for 5 min. The PCR product was cloned into the pEASY-Blunt vector (Transgen biotech, Beijing, China) and sequenced. The cloning of *EgFAD8* promoter region sequence has been published in our previous study [44].

**Table 1:** List of primers used in the study.

Primers	Sequence
EgFAD8-pYES2 F	5’-TAGGTACCATGGCGAGTTGGGTTCTATC-3’ (*Kpn*I)
EgFAD8-pYES2 R	5’-GCTCTAGATCAATCTGAGTTCTTCTGTGAAA-3’ (*Xba*I)
RT-EgFAD8 F	5’-TAACGGGAAACGGGTGAA-3’
RT-EgFAD8 R	5’-CCATCCAGTTATTGAGGTAGG-3’
RT-β-actin F	5’-TGGAAGCTGCTGGAATCCAT-3’
RT-β-actin R	5’-TCCTCCACTGAGCACAACGTT-3’

### Gene expression in *Saccharomyces*. *cerevisiae*

In order to generate a yeast expression construct, *EgFAD8* fragments were digested with *Kpn*I and *Xba*I from pEASY vector, and subcloned into pYES2 vector. The construct EgFAD8-pYES2 and the control pYES2 were separately transformed into INVSc1 cells using the polyethylene glycol/lithium acetate method [45,46]. Transformants were selected on synthetic complete medium lacking uracil (SC-U) supplemented with 2% (w/v) glucose. Three independent positive clones of each transformation were used for further expression studies.

Precultures were made in 2 mL SC-U medium with 2% (w/v) raffinose and 1% (w/v) tergitol NP-40 (Sigma, USA), and were grown overnight at 30°C. For induction gene expression, appropriate amount of precultures were inoculated into 20 mL SC-U medium containing 2% (w/v) galactose and 50 μM linoleic acid at an OD_600_ of 0.1. Yeast cultures were incubated for three days at 30°C under continuous agitation (250 rpm). Then the cells were harvested and washed twice with sterile deionized water before used for fatty acid analysis.

### Fatty acid analysis

Yeast cells were directly transmethylated by 2 mL of methanol containing 2.7% (v/v) H_2_SO_4_ at 80°C for 2 hours. Then, 3 mL of Hexane was added for recovering fatty acid methyl esters (FAMEs), and 2 mL of NaCl (2.5%, w/v) was used for phase separation. After centrifuge, the upper hexane phase was transferred to a fresh tube and applied for gas chromatography (GC) analysis.

### Exogenous application of low temperature and light treatments

Ten-day-old seedlings of the T3 generation of transgenic *Arabidopsis* were subjected to stress treatments. For low temperature and light treatments, transgenic plants were planted at 15°C and in dark, respectively, with other conditions are the same with the control (as mentioned in Materials). Quantitative analysis of GUS activity in each plant was carried out after treatments for 24h and 48h.

### Histochemical staining and fluorometric GUS assay

For histochemical GUS staining, various tissues of Arabidopsis were incubated in GUS assay buffer with 2 mM X-Gluc, 5 mM K_3_Fe(CN)_6_, 100 mM Na_3_PO_4_ (pH 7.0), 5 mM K_4_Fe(CN)_6_, 0.2% Triton X-100 and 10 mM EDTA at 37°C overnight under the darkness, and then cleared with 70% ethanol [47]. The samples were viewed by stereo-microscopy.

Quantitative analysis of GUS activity in transgenic Arabidopsis was determined according to Jefferson [47]. The protein concentrations of plant extracts were measured as descried by Bradford [48]. GUS activity was calculated as pmol 4-Methylumbelliferone (4-MU) per min per microgram protein. Three replicates were performed for each sample.

### Real-time quantitative PCR analysis

Total RNA from mature leaves and mesocarp at five developmental stages were extracted as described previously, and cDNAs was used for real-time quantitative PCR (RT-qPCR). EgFAD8 RT-PCR primers RT-EgFAD8 F/R (Table 1) were designed for RT-qPCR. The housekeeper gene β-actin (RT-β-actin F/R, Table 1) was used as an internal control for expression analysis. RT-qPCR was carried out using a CFX Connect^™^ Real-Time PCR Detection System (Bio-Rad Laboratories) and SYBR^®^ premix Ex Taq^™^ II (Tli RNaseH Plus) Kit (Takara, Tokyo, Japan) according to the manufacturer’s instructions. Expression was quantified as comparative threshold cycle (Ct) using 2^–ΔΔCt^ method [49]. Reactions were in triplicate including template-free and no-reverse-transcriptase negative controls.

## Results

### Cloning and bioinformatic analysis of *EgFAD8*

**Fig 1:**
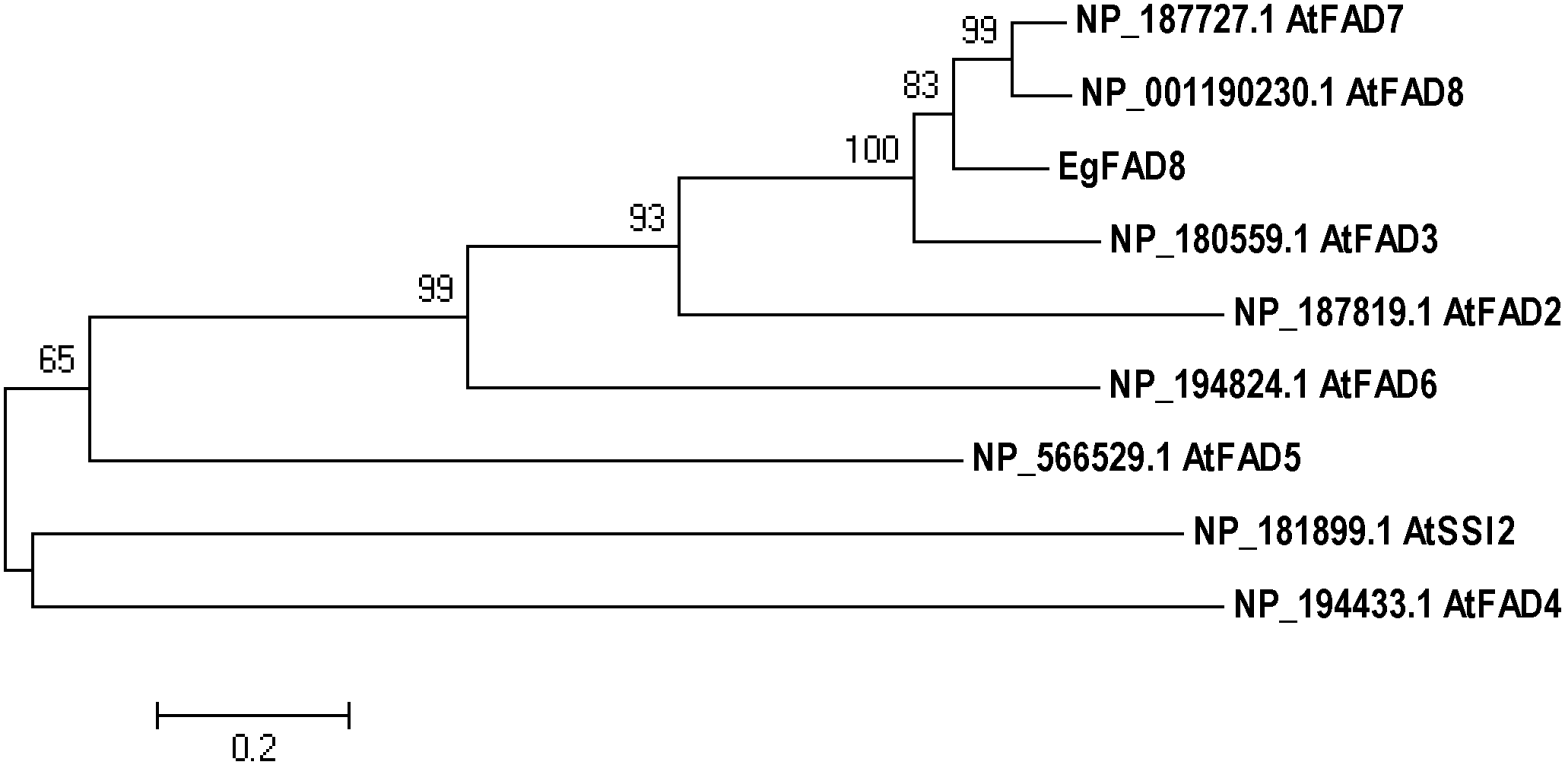
Phylogenetic analysis of EgFAD8 and fatty acid desaturases from *Arabidopsis thaliana*. A neighbor-joining tree was generated by MEGA5 with bootstrap analysis based on 1000 replications. Bootstrap values are shown at branch points. Scale bar indicates the number of substitutions per site.

The open reading frame (ORF) cDNA sequence (1362 bp) of a ω-3 fatty acid desaturase (designated as *EgFAD8*) was cloned from oil palm (*Elaeis guineensis* Jacq.) mesocarp based on that gene sequence from *Elaeis oleifera* (GenBank accession: EU057620.1) using RT-PCR. The nucleotide sequence is 100% identity with that from *Elaeis oleifera*. It encodes a 453-amino acid polypeptide with the conserved domain of ω-3 fatty acid desaturase (amino acids 1–443), which is predicted to be chloroplastic. The protein BLAST analysis of EgFAD8 showed that it shared high homology (more than 70%) with other members of the ω-3 desaturase enzyme family. Phylogenetic analysis of EgFAD8 with various fatty acid desaturases from model plant *Arabidopsis thaliana* revealed that EgFAD8 clusters with two chloroplastic ω-3 fatty acid desaturases AtFAD7 and AtFAD8, as well as the endoplasmic reticulum-type ω-3 fatty acid desaturase AtFAD3 (Fig 1).

### Expression pattern of *EgFAD8* in leaf and mesocarp during fruit development

**Fig 2:**
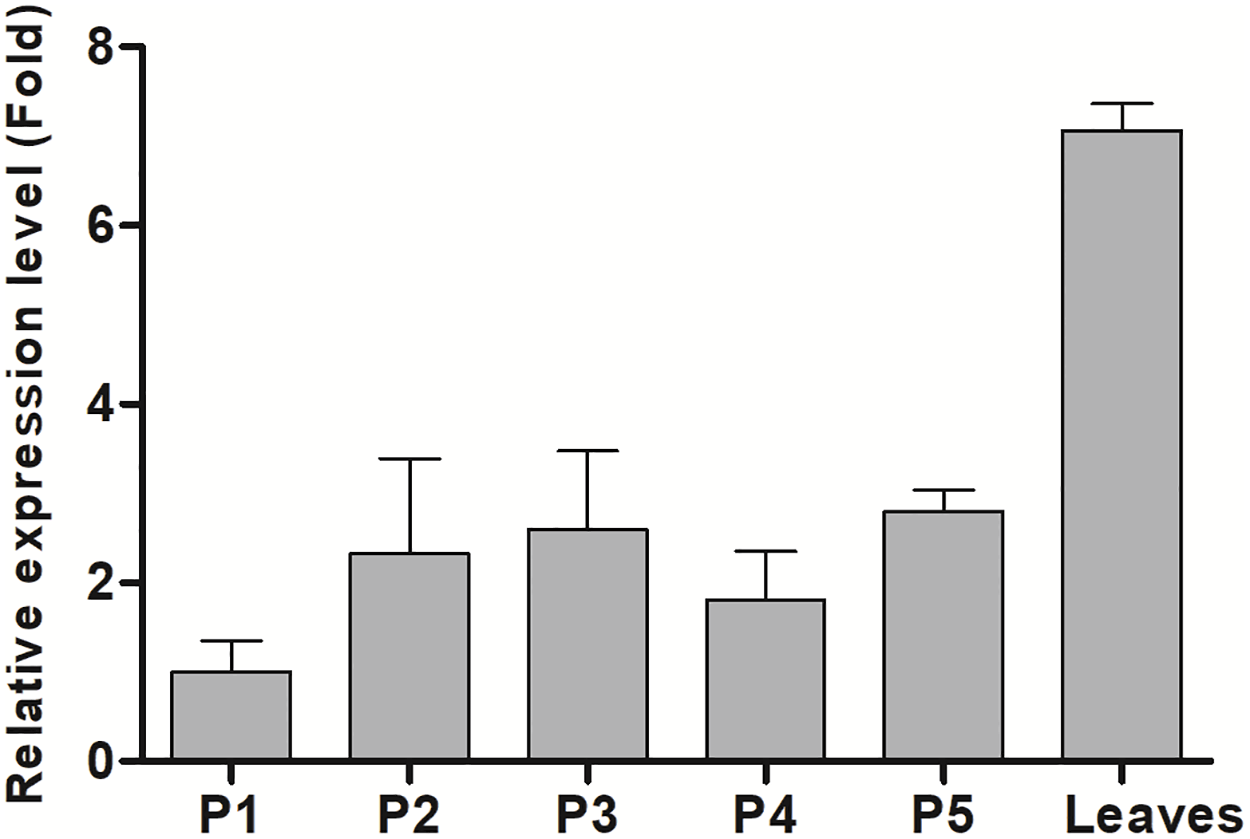
Expression analysis of *EgFAD8* in oil palm leaves and mesocarp at five different developmental stages. P1: mesocarp at 30–60 DAP; P2: mesocarp at 60–100 DAP; P3: mesocarp at 100–120 DAP; P4: mesocarp at 120–140 DAP; P5: mesocarp at 140–160 DAP. DAP: days after pollination.

The transcript levels of *EgFAD8* in leaf and mesocarp at five different developmental stages were determined by RT-qPCR using *β*-actin as an internal control. The results showed that the transcript level of *EgFAD8* in mesocarp was quite flat during ripening with a slight increase at the beginning (from P1 to P2, Fig 2). Interestingly, the expression level of *EgFAD8* in mature leaves was at least twice that in mescoarp (Fig 2).

### Heterologous expression of *EgFAD8* in yeast could produce 18:3 using exogenous 18:2

**Fig 3:**
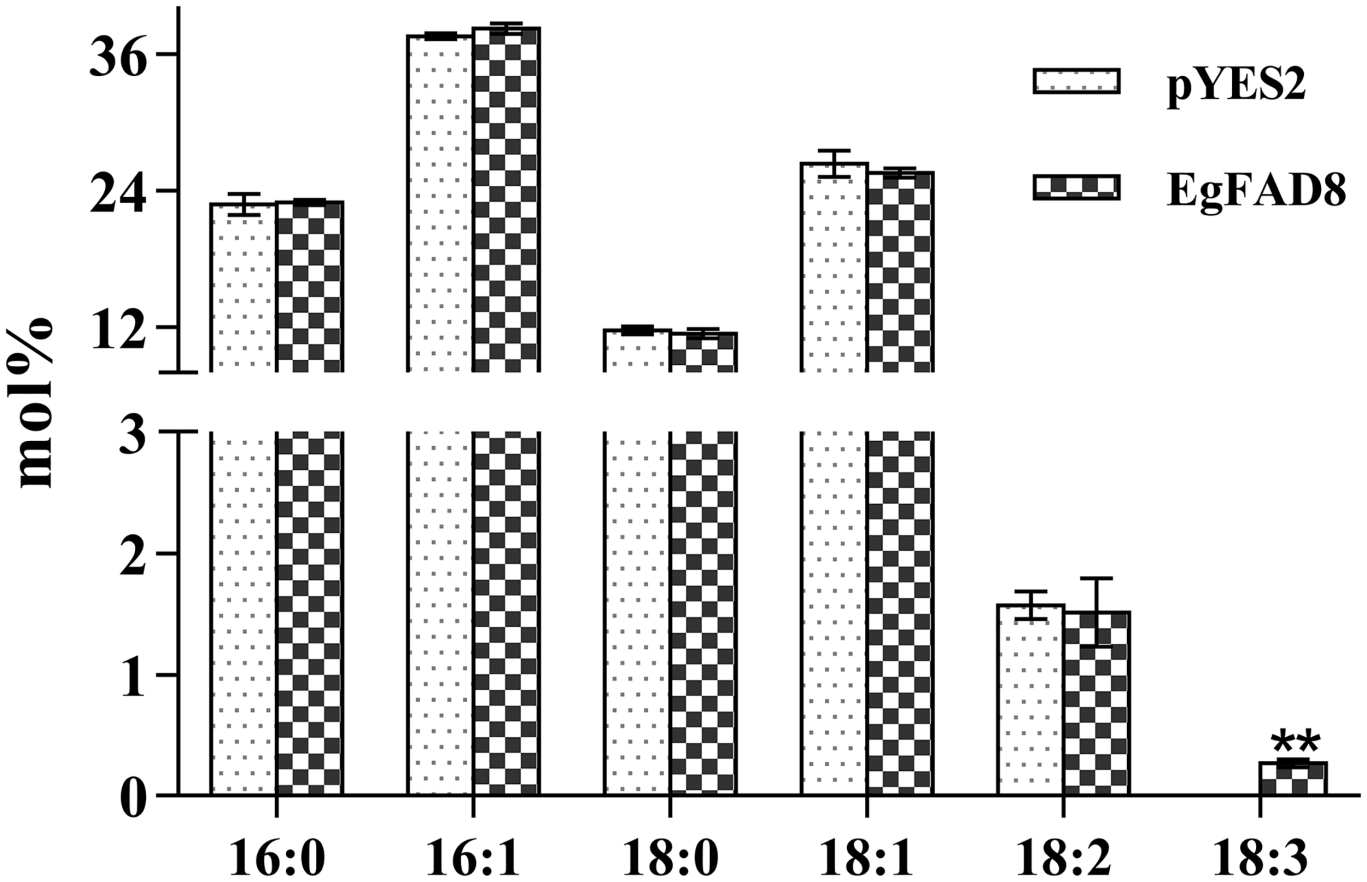
Total fatty acid composition of transformed yeast with *EgFAD8* or pYES2. All experiments were carried out in triplicate. Asterisks indicate statistically significant differences compared with the control (Student’s *t* test: **, p < 0.01). 16:0, palmitic acid; 16:1, palmitoleic acid; 18:0, stearic acid; 18:1, oleic acid; 18:2, linoleic acid; 18:3,alpha linoleic acid.

The activity of EgFAD8 was determined by heterologous expression in *S*. *cerevisiae* supplemented with linoleic acid as substrate, which is absent in yeast. Yeast transformed with empty vector (pYES2) was used as negative control. The total fatty acid composition of yeast transformed with *EgFAD8* or pYES2 was detected and analyzed. The result showed that yeast transformed with *EgFAD8* produced a new fatty acid species 18:3 ω-3 (about 0.27 mol%) in yeast, but not presented in the negative control (Fig 3). Meanwhile, there was no obvious difference in the levels of other fatty acids between yeast transformed with *EgFAD8* and the negative control pYES2 (Fig 3). Therefore, the expression of *EgFAD8* in yeast could produce 18:3 using the supplemented 18:2.

### The activity of *EgFAD8* promoter response to growth environment treatments

**Fig 4:**
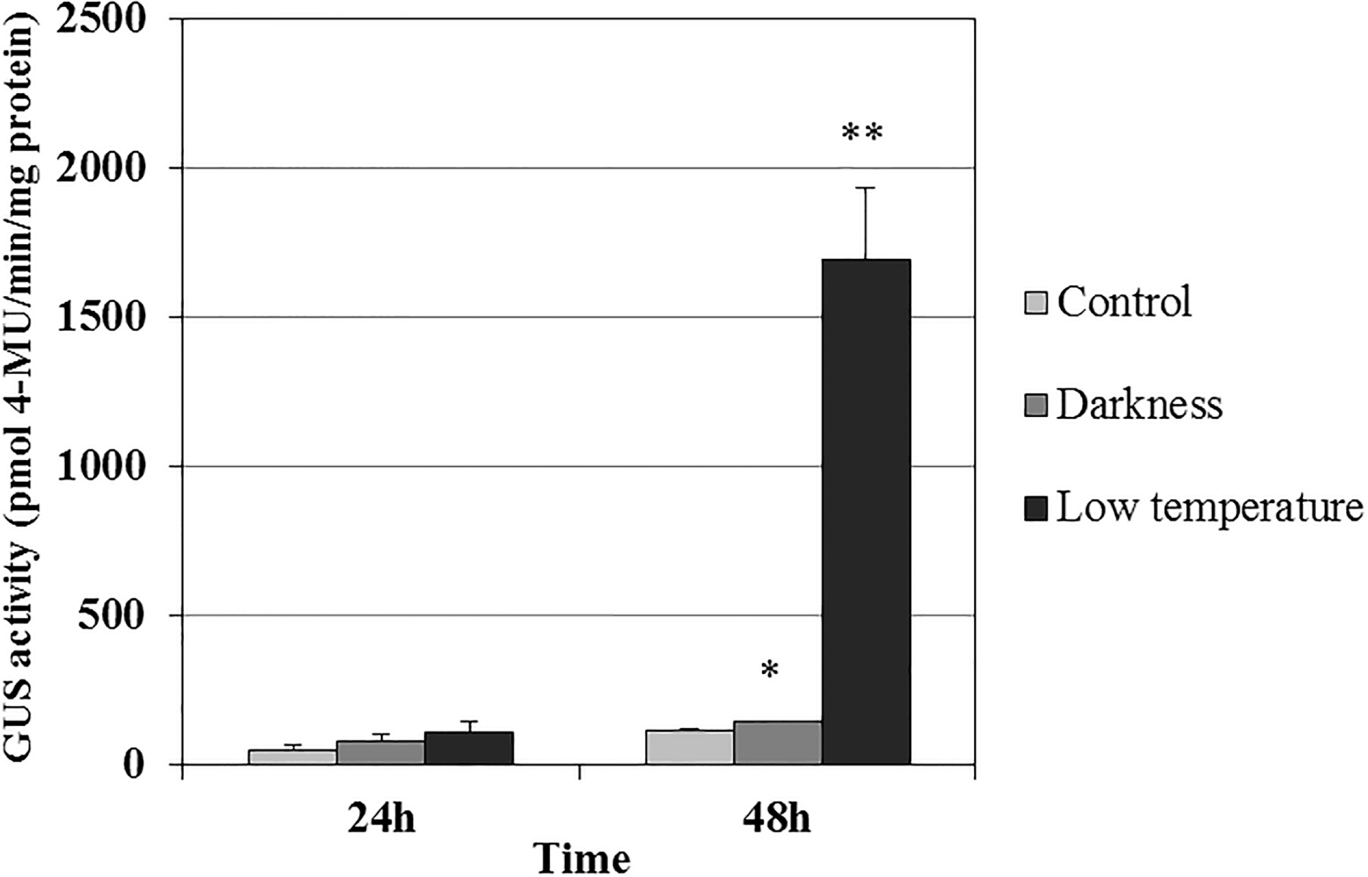
Quantification of GUS activity in leaves of transgenic Arabidopsis transformed with *ProEgFAD8*::*GUS* under the treatment of darkness or low temperature (15°C) for 24h and 48h.

Promoter prediction analysis suggested that the *EgFAD8* promoter contained some stress-responsive elements, including light-responsive elements. Therefore, further work was carried out to investigate whether it was induced under various stress conditions, such as cold and dark. The *ProEgFAD8*::*GUS* transgenic Arabidopsis T3 lines were cultivated at 15°C and in the dark, respectively, with other conditions are the same with the control. The GUS activity of transgenic plants under the darkness or low temperature treatment was no obvious difference with that of the control after 24 hours treatment (Fig 4). However, 48 hours treatment later, the GUS activity of the transgenic plants under the darkness treatment showed obvious increase by 26% when compared with that of the control, and the transgenic plants under the low temperature treatment exhibited the extraordinarily significant increase of GUS activity by about 14-fold comparing with the control (Fig 4).

## Discussion

In this study, we identified and characterized the first chloroplast ω-3 fatty acid desaturase (*EgFAD8*) from oil palm. It exhibited the capability of synthesizing 18:3 ω-3 when heterologously expressed in yeast with the exogenous supply of linoleic acid. However, there are three ω-3 fatty acid desaturases in Arabidopsis: the ER-localized FAD3 and two chloroplastic FAD7 and FAD8. When compared with fatty acid desaturases from Arabidopsis, EgFAD8 showed the highest similarity with the temperature-sensitive chloroplastic ω-3 fatty acid desaturase and AtFAD8 (data not shown). Moreover, the phylogenetic analysis of EgFAD8 revealed that it is grouped with AtFAD7 and AtFAD8 from Arabidopsis, but with more close relation with AtFAD8 (Fig 1). Therefore, the bioinformatic analysis of EgFAD8 indicated that the *EgFAD8* gene might encode a plastidial FAD8, which could be induced by low temperature [16]. Nevertheless, more experimental evidence should be shown to support the results from sequence analysis although FAD8 sequences are highly conserved in plants [17].

Furthermore, the expression pattern in oil palm mesocarp at five developmental stages and leaves was also detected to see whether the expression level of *EgFAD8* is correlated with oil deposition in oil palm mesocarp. While, we can see *EgFAD8* was predominately expressed in leaves, at least twice-fold as that of mesocarp. Moreover, the expression level of *EgFAD8* remains still during fruit ripening with a slight increase at the beginning. The majority of oil deposition in oil palm mesocarp occurs at the late stages (from phase 3 to phase 5) [32,35]. Thus, the expression of *EgFAD8* is not correlated with oil accumulation in mesocarp. Similar patterns have been seen in the expression of *FAD8* in other plant species [11,13,17]. The *GmFAD8* genes from soybean were constitutively expressed in all vegetative tissues (roots, stems, mature leaves and flowers) analyzed at optimal growth temperatures [11,13]. Like *EgFAD8*, the plastidial *PfrFAD7* genes from *Perilla frutescens var*. *frutescens* were expressed at low levels during all developmental stages of seeds, when compared with expression levels in leaves [17]. Therefore, this result is also another proof to support *EgFAD8* encodes a plastidial ω-3 fatty acid desaturase, rather than the microsomal ω-3 fatty acid desaturase.

In order to study the *in vivo* function of EgFAD8, it has been successfully expressed in *S*. *cerevisiae*. However, there is no linoleic acid (18:2) produced in yeast. So the exogenous supply of 18:2 is needed for studying the activity of ω-3 fatty acid desaturase. The result from GC analysis indicated that the *EgFAD8* transformed yeast cells could produce slight amounts of 18:3 using the exogenous 18:2. At least, this result confirmed the *in vivo* activity of ω-3 fatty acid desaturase encoded by *EgFAD8* from oil palm. While, the amount of 18:3 produced by the *EgFAD8* transformed yeast is very limited. There might be three possible reasons as follows: first, the *EgFAD8* gene is likely to encode a chloroplast ω-3 fatty acid desaturase, rather than the ER-localized desaturase; thus it could not participate in the synthesis of storage oil in oil palm mesocarp. Likewise, when expressed in yeast cells, EgFAD8 might just be involved in the desaturation of galactolipids in the plastid. Second, EgFAD8 has been predicted to be temperature-sensitive desaturase by bioinformatic analysis. The yeast growth condition at 30 °C might not intrigue the large expression of transformed gene *EgFAD8*. Third, the exogenous supply of 18:2 might be a bit unhealthy or toxic for yeast cell. So, the coexpression of ω-3 fatty acid desaturase with ω-6 fatty acid desaturase in yeast might be a better solution than feeding with 18:2 in the yeast culture. However, the *JcFAD7* from *Jatropha curcas* also showed the similar result when expressed in yeast, producing about 2.5% of 18:3 in transformed cells [24]. Therefore, the *EgFAD8* gene from oil palm encodes a ω-3 fatty acid desaturase.

Furthermore, in order to elucidate the physiological role of EgFAD8, we also detected whether the *EgFAD8* promoter could be affected by the environmental stress including low-temperature and darkness treatments. In our previous study, the activity of *EgFAD8* promoter in transgenic Arabidopsis has been characterized (S1 Fig) [44]. The GUS staining results indicated that the *EgFAD8* promoter has the expected capability to drive transgene expression in all tissues tested, except with very weak expression in roots (S1 Fig). Thus, we used the *ProEgFAD8*::*GUS* transgenic plants for the stress treatment analysis, since the EgFAD8 promoter is constitutively active in transgenic Arabidopsis. The results revealed that the GUS activity of the transgenic plants under the darkness and low temperature treatment was increased by 26% and 14-folds after 48-hour treatment when compared with that of the control, respectively (Fig 4). So the *EgFAD8* gene from oil palm could be significantly induced by low temperature and slightly induced by darkness. This is consistent with the induction expression of *AtFAD8* from Arabidopsis when treated at 4 °C [16,50]. Similarly, the *PoleFAD8* transcript level of *Portulaca oleracea* L. also increased significantly after treatment at 5 °C [51]. As expected, this result also evidenced that the chloroplastic *EgFAD8* from oil palm is cold-inducible. On the other hand, lots of light regulatory elements were predicted in the promoter of *EgFAD8* by PlantCARE [44]. The result of the darkness treatment on transgenic plants is also consistent with the bioinformatic prediction. The darkness could slightly induce the expression of *EgFAD8*. While, the mechanism that the light regulates the expression of *FAD8* still remains obscure. Our hypothesis is that more chloroplasts are needed for fatty acid synthesis in the dark than in the light, since the rate of fatty acid synthesis in leaves is six-fold higher in the light than in the dark [2]. Thus, to maintain the constitutive function, more chloroplasts are synthesized in the leave cells, and the expression level of genes encoding the corresponding enzymes involved in the synthesis of chloroplast might be up-regulated. FAD8 is responsible for the polyunsaturated components of chloroplast membranes [16]. However, the related mechanism needs to be elucidated.

In conclusion, we have isolated and identified a plastidial ω-3 fatty acid desaturase (*EgFAD8*) from oil palm. The *in vivo* function of EgFAD8 has been confirmed by heterologously expression in yeast, with showing the conversion of linoleic to linolenic acid. Moreover, the expression profile of *EgFAD8* also revealed that it mainly involved in the synthesis of chloroplast membrane, unlike ER-localized ω-3 fatty acids that is tightly correlated with the oil accumulation in seeds or fruits [1,20,25]. Furthermore, the characterization of the *EgFAD8* promoter indicated that the *EgFAD8* gene could be induced by low temperature and darkness. Overall, these results will be helpful for understanding the function and regulation of plastidial ω-3 fatty acid desaturases in higher plants.

## Supporting information

S1 Fig Histochemical GUS staining of transgenic Arabidopsis under the control of EgFAD8 promoter. The results from transgenic Arabidopsis: A1-A5; the untransformed Arabidopsis were used as the negative control: B1-B5. 1: three-week-old seedling; 2: leaves; 3: roots; 4: flowers and stems; 5: silique coats.
(TIF)

## References

[1] Hernández ML, Sicardo MD, Martínez-Rivas JM. Differential Contribution of Endoplasmic Reticulum and Chloroplast ω-3 Fatty Acid Desaturase Genes to the Linolenic Acid Content of Olive (*Olea europaea*) Fruit. Plant Cell Physiol. 2016;57: 138–151. doi: 10.1093/pcp/pcv159 26514651 10.1093/pcp/pcv159

[2] Ohlrogge J, Browse J. Lipid Biosynthesis. PLANT CELL ONLINE. 1995;7: 957–970. doi: 10.1105/tpc.7.7.957 7640528 10.1105/tpc.7.7.957 PMC160893

[3] Crawford MA. The role of essential fatty acids and prostaglandins. Postgrad Med J. 1980;56: 557–562. doi: 10.1136/pgmj.56.658.557 7465459 10.1136/pgmj.56.658.557 PMC2425954

[4] Damude HG, Zhang H, Farrall L, Ripp KG, Tomb J-F, Hollerbach D, et al Identification of bifunctional Δ12/ω3 fatty acid desaturases for improving the ratio of ω3 to ω6 fatty acids in microbes and plants. Proc Natl Acad Sci. 2006;103: 9446–9451. doi: 10.1073/pnas.0511079103 16763049 10.1073/pnas.0511079103 PMC1480427

[5] Baker EJ, Miles EA, Burdge GC, Yaqoob P, Calder PC. Metabolism and functional effects of plant-derived omega-3 fatty acids in humans. Prog Lipid Res. 2016;64: 30–56. doi: 10.1016/j.plipres.2016.07.002 27496755 10.1016/j.plipres.2016.07.002

[6] Calder PC. n-3 Polyunsaturated Fatty Acids and Inflammation : From Molecular Biology to the Clinic. Inflammation. 2003;38: 343–352. 10.1007/s11745-003-1068-y PMC7101988 12848278

[7] Simon E, Bardet B, Grégoire S, Acar N, Bron AM, Creuzot-Garcher CP, et al Decreasing dietary linoleic acid promotes long chain omega-3 fatty acid incorporation into rat retina and modifies gene expression. Exp Eye Res. 2011;93: 628–635. doi: 10.1016/j.exer.2011.07.016 21821023 10.1016/j.exer.2011.07.016

[8] Hamilton ML, Haslam RP, Napier JA, Sayanova O. Metabolic engineering of Phaeodactylum tricornutum for the enhanced accumulation of omega-3 long chain polyunsaturated fatty acids. Metab Eng. Elsevier; 2014;22: 3–9. doi: 10.1016/j.ymben.2013.12.003 24333273 10.1016/j.ymben.2013.12.003 PMC3985434

[9] Shahidi F. Omega-3 fatty acids and marine oils in cardiovascular and general health: A critical overview of controversies and realities. J Funct Foods. 2015;19: 797–800. doi: 10.1016/j.jff.2015.09.038

[10] Browse J, Somerville C. Glycerolipid Synthesis: Biochemistry and Regulation. Annu Rev Plant Physiol Plant Mol Biol. 1991;42: 467–506. doi: 10.1146/annurev.pp.42.060191.002343

[11] Andreu V, Lagunas B, Collados R, Picorel R, Alfonso M. The *GmFAD7* gene family from soybean: Identification of novel genes and tissue-specific conformations of the FAD7 enzyme involved in desaturase activity. J Exp Bot. 2010;61: 3371–3384. doi: 10.1093/jxb/erq158 20547564 10.1093/jxb/erq158 PMC2905200

[12] Li-Beisson Y, Shorrosh B, Beisson F, Andersson MX, Arondel V, Bates PD, et al Acyl-lipid metabolism. Arabidopsis Book. 2013;11: e0161 doi: 10.1199/tab.0161 23505340 10.1199/tab.0161 PMC3563272

[13] Román, Chiappetta A, Bruno L, Bitonti MB. Contribution of the different omega-3 fatty acid desaturase methylation and chromatin patterning genes to the cold response in soybean. J Exp Bot. 2012;63: 695–709.22058406

[14] Rajwade A V., Kadoo NY, Borikar SP, Harsulkar AM, Ghorpade PB, Gupta VS. Differential transcriptional activity of SAD, FAD2 and FAD3 desaturase genes in developing seeds of linseed contributes to varietal variation in α-linolenic acid content. Phytochemistry. Elsevier Ltd; 2014;98: 41–53. doi: 10.1016/j.phytochem.2013.12.002 24380374 10.1016/j.phytochem.2013.12.002

[15] Murphy DJ, Piffanelli P. Fatty acid desaturases: structure, mechanism and regulation Plant lipid biosynthesis: fundamentals and agricultural applications. Cambridge: Cambridge University Press; 1998.

[16] Gibson S, Arondel V, Iba K, Somerville C. Cloning of a temperature-regulated gene encoding a chloroplast omega-3 desaturase from *Arabidopsis thaliana*. Plant Physiol. 1994;106: 1615–1621. doi: 10.1104/pp.106.4.1615 7846164 10.1104/pp.106.4.1615 PMC159705

[17] Lee KR, Lee Y, Kim EH, Lee SB, Roh KH, Kim JB, et al Functional identification of oleate 12-desaturase and ω-3 fatty acid desaturase genes from *Perilla frutescens var*. *frutescens*. Plant Cell Rep. 2016;35: 2523–2537. doi: 10.1007/s00299-016-2053-4 27637203 10.1007/s00299-016-2053-4

[18] Yadav NS, Wierzbicki A, Aegerter M, Caster CS, Pérez-Grau L, Kinney AJ, et al Cloning of higher plant omega-3 fatty acid desaturases. Plant Physiol. 1993;103: 467–476. doi: 10.1104/pp.103.2.467 8029334 10.1104/pp.103.2.467 PMC159005

[19] Iba K, Gibson S, Nishiuchis T, Fuse T, Nishimuras M, Arondelll V, et al A gene encoding a chloroplast ω-3 fatty acid desaturase complements alterations in fatty acid desaturation and chloroplast copy number of the fad7 mutant of *Arabidopsis thaliana*. J Biol Chem. 1993;268: 24099–24105. 8226956

[20] Bilyeu KD, Palavalli L, Sleper DA, Beuselinck PR. Three microsomal omega-3 fatty-acid desaturase genes contribute to soybean linolenic acid levels. Crop Sci. 2003;43: 1833–1838. doi: 10.2135/cropsci2003.1833

[21] Berberich T, Harada M, Sugawara K, Kodama H, Iba K, Kusano T. Two maize genes encoding ω-3 fatty acid desaturase and their differential expression to temperature. 1998; 297–306. 10.1023/a:1005993408270 9484441

[22] Banilas G, Nikiforiadis A, Makariti I, Moressis A, Hatzopoulos P. Discrete roles of a microsomal linoleate desaturase gene in olive identified by spatiotemporal transcriptional analysis. Tree Physiol. 2007;27: 481–490. doi: 10.1093/treephys/27.4.481 17241990 10.1093/treephys/27.4.481

[23] Guan L, Wu W, Hu B, Li D, Chen J, Hou K, et al Devolopmental and growth temperature regulation of omega-3 fatty acid desaturase genes in safflower (*Carthamus tinctorius* L.). 2014;13: 6623–6637. 10.4238/2014.August.28.7 25177943

[24] Guo L, Qing R, He W, Xu Y, Tang L, Wang S, et al Identification and characterization of a plastidial ω3-fatty acid desaturase gene from Jatropha curcas. Chinese J Appl Environ Biol. 2008;14: 469–474.

[25] Vrinten P, Hu Z, Munchinsky M-A, Rowland G, Qiu X. Two FAD3 Desaturase Genes Control the Level of Linolenic Acid in Flax Seed. Plant Physiol. 2005;139: 79–87. doi: 10.1104/pp.105.064451 16113219 10.1104/pp.105.064451 PMC1203359

[26] Kodama H, Hamada T, Horiguchi G, Nishimura M, Iba K. Genetic Enhancement of Cold Tolerance by Expression of a Gene for Chloroplast [omega]-3 Fatty Acid Desaturase in Transgenic Tobacco. Plant Physiol. 1994;105: 601–605. 12232227 10.1104/pp.105.2.601 PMC159399

[27] Iba K. Acclimative response to temperature stress in higher plants: approaches of gene engineering for temperature tolerance. Annu Rev Plant Biol. 2002;53: 225–245. doi: 10.1146/annurev.arplant.53.100201.160729 12221974 10.1146/annurev.arplant.53.100201.160729

[28] Dominguez T, Hernandez ML, Pennycooke JC, Jimenez P, Martinez-Rivas JM, Sanz C, et al Increasing ω-3 Desaturase Expression in Tomato Results in Altered Aroma Profile and Enhanced Resistance to Cold Stress. Plant Physiol. 2010;153: 655–665. doi: 10.1104/pp.110.154815 20382895 10.1104/pp.110.154815 PMC2879794

[29] D’Angeli S, Matteucci M, Fattorini L, Gismondi A, Ludovici M, Canini A, et al OeFAD8, OeLIP and OeOSM expression and activity in cold-acclimation of *Olea europaea*, a perennial dicot without winter-dormancy. Planta. 2016;243: 1279–1296. doi: 10.1007/s00425-016-2490-x 26919986 10.1007/s00425-016-2490-x PMC4837226

[30] Szymanski J, Brotman Y, Willmitzer L, Cuadros-Inostroza A. Linking Gene Expression and Membrane Lipid Composition of Arabidopsis. Plant Cell. 2014;26: 915–928. doi: 10.1105/tpc.113.118919 24642935 10.1105/tpc.113.118919 PMC4001401

[31] Lagunas B, Román Á, Andreu V, Picorel R, Alfonso M. A temporal regulatory mechanism controls the different contribution of endoplasmic reticulum and plastidial ω-3 desaturases to trienoic fatty acid content during leaf development in soybean (*Glycine max cv Volania*). Phytochemistry. 2013;95: 158–167. doi: 10.1016/j.phytochem.2013.07.012 23928132 10.1016/j.phytochem.2013.07.012

[32] Bourgis F, Kilaru A, Cao X, Ngando-ebongue G-F, Drira N, Ohlrogge JB, et al Comparative transcriptome and metabolite analysis of oil palm and date palm mesocarp that differ dramatically in carbon partitioning. Proc Natl Acad Sci U S A. 2011;108: 12527–12532. doi: 10.1073/pnas.1106502108 21709233 10.1073/pnas.1106502108 PMC3145713

[33] Bazmi AA, Zahedi G, Hashim H. Progress and challenges in utilization of palm oil biomass as fuel for decentralized electricity generation. Renew Sustain Energy Rev. 2011;15: 574–583. doi: 10.1016/j.rser.2010.09.031

[34] Dislich C, Keyel AC, Salecker J, Kisel Y, Meyer KM, Auliya M, et al A review of the ecosystem functions in oil palm plantations, using forests as a reference system. Biol Rev. 2016;49 doi: 10.1111/brv.12295 27511961 10.1111/brv.12295

[35] Dussert S, Guerin C, Andersson M, Joet T, Tranbarger TJ, Pizot M, et al Comparative Transcriptome Analysis of Three Oil Palm Fruit and Seed Tissues That Differ in Oil Content and Fatty Acid Composition. Plant Physiol. 2013;162: 1337–1358. doi: 10.1104/pp.113.220525 23735505 10.1104/pp.113.220525 PMC3707537

[36] Corley RHV, Tinker PB, editors. The Oil Palm [Internet]. Oxford, UK: Blackwell Science Ltd; 2003 doi: 10.1002/9780470750971

[37] Ebong PE, Owu DU, Isong EU. Influence of palm oil (*Elaesis guineensis*) on health. Plant Foods Hum Nutr. 1999;53: 209–222. doi: 10.1023/A:1008089715153 10517280 10.1023/a:1008089715153

[38] Sun R, Gao L, Yu X, Zheng Y, Li D, Wang X. Identification of a Δ12 fatty acid desaturase from oil palm (*Elaeis guineensis* Jacq.) involved in the biosynthesis of linoleic acid by heterologous expression in Saccharomyces cerevisiae. Gene. Elsevier B.V.; 2016;592: 21–26. doi: 10.1016/j.gene.2016.06.039 27370696 10.1016/j.gene.2016.06.039

[39] Jin Y, Yuan Y, Gao L, Sun R, Chen L, Li D, et al Characterization and Functional Analysis of a Type 2 Diacylglycerol Acyltransferase (*DGAT2*) Gene from Oil Palm (*Elaeis guineensis* Jacq.) Mesocarp in Saccharomyces cerevisiae and Transgenic Arabidopsis thaliana. Front Plant Sci. 2017;8: 1–10. 29089956 10.3389/fpls.2017.01791 PMC5651047

[40] Larkin MA, Blackshields G, Brown NP, Chenna R, Mcgettigan PA, McWilliam H, et al Clustal W and Clustal X version 2.0. Bioinformatics. 2007;23: 2947–2948. doi: 10.1093/bioinformatics/btm404 17846036 10.1093/bioinformatics/btm404

[41] Tamura K, Peterson D, Peterson N, Stecher G, Nei M, Kumar S. MEGA5: molecular evolutionary genetics analysis using maximum likelihood, evolutionary distance, and maximum parsimony methods. Mol Biol Evol. Oxford University Press; 2011;28: 2731–9. doi: 10.1093/molbev/msr121 21546353 10.1093/molbev/msr121 PMC3203626

[42] Lescot M, Déhais P, Thijs G, Marchal K, Moreau Y, Van de Peer Y, et al PlantCARE, a database of plant cis-acting regulatory elements and a portal to tools for in silico analysis of promoter sequences. Nucleic Acids Res. 2002;30: 325–327. doi: 10.1093/nar/30.1.325 11752327 10.1093/nar/30.1.325 PMC99092

[43] Li D, Fan Y. Extraction and quality analysis of total RNA from pulp ofcoconut (*Cocos nucifera* L.). Mol Plant Breed (in Chinese). 2007;5: 883–886.

[44] Wang L, Gao L, Zheng Y, Li D. Cloning and expression tissue-specific analiysis of fatty acid desaturase gene ω3 promoter from oil palm. Mol Plant Breed (in Chinese). 2016;14: 570–577.

[45] Gietz RD, Schiestl RH, Willems AR, Woods RA. Studies on the transformation of intact yeast cells by the LiAc/SS-DNA/PEG procedure. Yeast. John Wiley & Sons, Ltd.; 1995;11: 355–360. doi: 10.1002/yea.320110408 7785336 10.1002/yea.320110408

[46] Ito H, Fukuda Y, Murata K. Transformation of intact yeast cells treated with alkali Transformation of Intact Yeast Cells Treated with Alkali Cations. 1983;153: 166–168. 10.1128/jb.153.1.163-168.1983 PMC217353 6336730

[47] Jefferson RA, Kavanagh TA, Bevan MW. GUS fusions: beta-glucuronidase as a sensitive and versatile gene fusion marker in higher plants. EMBO J. 1987;6: 3901–7. 3327686 10.1002/j.1460-2075.1987.tb02730.x PMC553867

[48] Bradford MM. A rapid and sensitive method for the quantiWcation of microgram quantities of protein utilizing the principle of protein—dye binding. Anal Biochem. 1976;72: 248–254. doi: 10.1016/0003-2697(76)90527-3 942051 10.1016/0003-2697(76)90527-3

[49] Livak KJ, Schmittgen TD. Analysis of Relative Gene Expression Data Using Real-Time Quantitative PCR and the 2−ΔΔCT Method. Methods. 2001;25: 402–408. doi: 10.1006/meth.2001.1262 11846609 10.1006/meth.2001.1262

[50] Kargiotidou A, Deli D, Galanopoulou D, Tsaftaris A, Farmaki T. Low temperature and light regulate delta 12 fatty acid desaturases (*FAD2*) at a transcriptional level in cotton (*Gossypium hirsutum*). J Exp Bot. 2008;59: 2043–2056. doi: 10.1093/jxb/ern065 18453533 10.1093/jxb/ern065 PMC2413273

[51] Teixeira MC, Carvalho IS, Brodelius M. ω-3 Fatty acid desaturase genes isolated from purslane (*portulaca oleracea* L.): Expression in different tissues and response to cold and wound stress. J Agric Food Chem. 2010;58: 1870–1877. doi: 10.1021/jf902684v 20070085 10.1021/jf902684v

