# Supplementary figures and images for: Identification and characterization of a plastidial ω-3 fatty acid desaturase EgFAD8 from oil palm (Elaeis guineensis Jacq.) and its promoter response to light and low temperature
Source: PLoS One. 2018 Apr 26;13(4):e0196693. doi: 10.1371/journal.pone.0196693 (PMC5919639; doi:10.1371/journal.pone.0196693)

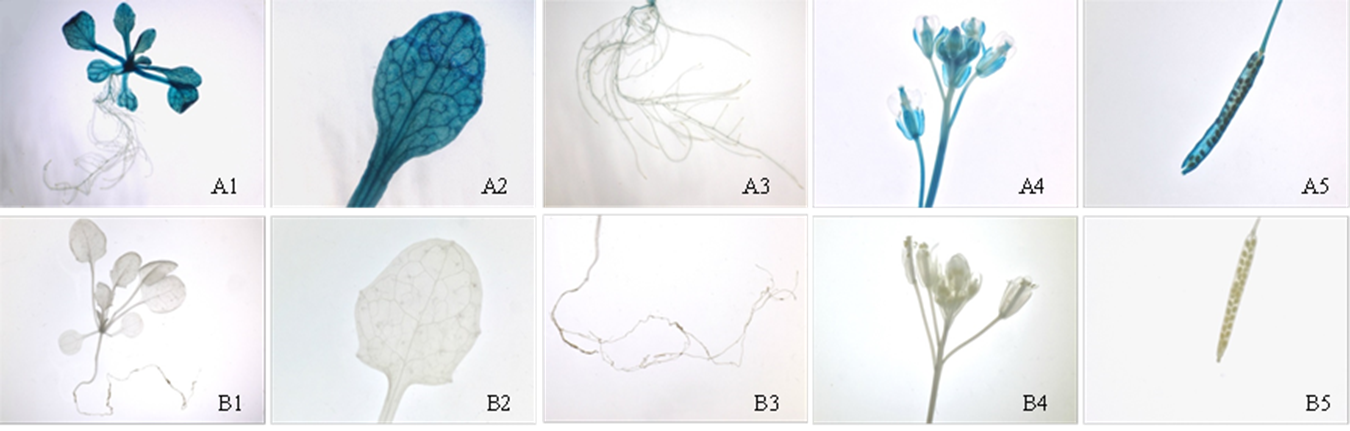

Supplement: S1 Fig — The results from transgenic Arabidopsis: A1-A5; the untransformed Arabidopsis were used as the negative control: B1-B5. 1: three-week-old seedling; 2: leaves; 3: roots; 4: flowers and stems; 5: silique coats. (TIF) [file pone.0196693.s001.tif]
